# Supplementary material for: Duality between predictability and reconstructability in complex systems
Source: Nat Commun. 2024 May 25;15:4478. doi: 10.1038/s41467-024-48020-x (PMC11127975; doi:10.1038/s41467-024-48020-x)
Supplement: Supplementary file 1 — Supplementary Information [file 41467_2024_48020_MOESM1_ESM.pdf]

Duality between predictability and reconstructability in complex systems
— Supplementary Information —

Charles Murphy,* Vincent Thibeault, Antoine Allard, and Patrick Desrosiers†

(Dated: February 29, 2024)

Contents

I. Duality between prediction and reconstruction performance 2

II. Analytical solutions for simple systems 4
  A. Two graphs and three time series 4
  B. Markov process on a single node graph 5

III. Predictability and reconstructability of deterministic dynamics 6

IV. Proof of the θ-duality between extrema 13

V. Proof of the monotonicity of $I(\mathbf{X};G)$ with T 14

VI. Proof of the existence of continuous extensions of uncertainty coefficients with T 16

VII. Numerical analysis of the past-dependent measures 18

VIII. Evaluation and bias of the mutual information in large systems 20
  A. Effect of biased mutual information over the uncertainty coefficients 20
  B. Upper bound: The stepping-stone algorithm 21
  C. Numerical results 22

IX. Numerical estimation of the phase transition thresholds 24

* charles.murphy.1@ulaval.ca
† patrick.desrosiers@phy.ulaval.ca

Supplementary Note I: Duality between prediction and reconstruction performance

Since $U(\mathbf{X}\,|\,G)$ is a relative measure of information, where both its denominator and numerator vary with J, we need to choose an adequate performance measure in order to make a fair comparison. Note that this is not a problem for $U(G\,|\,\mathbf{X})$ only because its denominator remains constant with J: If we wanted to investigate the duality with respect to, say, the number of edges, a similar treatment would have to be done with reconstruction performance measures as well. That being said, we compare $U(\mathbf{X}\,|\,G)$ with the relative MAE (RMAE), i.e., the MAE normalized by the MAE between the $\mathbf{X}$ itself and the probabilities of graph-independent model:

$$\mathrm{RMAE}(\hat{\mathbf{P}}, \mathbf{P}^*) = \frac{\mathrm{MAE}(\hat{\mathbf{P}}, \mathbf{P}^*)}{\mathbb{E}\left[\mathrm{MAE}(\hat{\mathbf{P}}, \mathbf{X})\right]}\,, \tag{1}$$

where

$$\mathrm{MAE}(\mathbf{X}, \mathbf{Y}) = \frac{1}{NT}\sum_{i=1}^{N}\sum_{t=1}^{T}|X_{i,t} - Y_{i,t}|\,, \tag{2}$$

and where we assume that $\mathbf{X}$ is generated with the entries of the probability matrix $\mathbf{P}^*$ (see Material and Methods, Section B.1 for the details, e.g., on $\hat{\mathbf{P}}$). The denominator measures the absolute error of the graph-independent model, which is related with $H(\mathbf{X})$, while the numerator measure the distance between the graph-dependent and graph-independent models, which is analoguous to $I(\mathbf{X}; G)$. Furthermore, the RMAE share another desirable property with $U(\mathbf{X}\,|\,G)$.

Lemma 1. *If $\mathbf{X}$ be a NT-dimensional Bernouilli random variable with a parameter matrix $\mathbf{P}^*$ and the elements of $\hat{\mathbf{P}}$ are in $(0,1)$, the RMAE between $\hat{\mathbf{P}}$ and $\mathbf{P}^*$ defined in Eq. (1) is bounded in the unit interval $[0,1]$.*

Proof. First, we have that $\mathbf{X} = (X_{i,t})_{i,t}$ with $X_{i,t} \in \{0,1\}$ for all $i \in \{1, ..., N\}$, $t \in \{1, ..., T\}$ and $P(X_{i,t} = 1) = P^*_{i,t}$, $P(X_{i,t} = 0) = 1 - P^*_{i,t}$. As expected for a Bernouilli random variable, we get

$$\mathbb{E}\left[X_{i,t}\right] = \sum_{x_{i,t}\in\{0,1\}} P(X_{i,t} = x_{i,t})\, x_{i,t} = P^*_{i,t} \tag{3}$$

and consequently, $\mathbf{P}^* = \mathbb{E}\left[\mathbf{X}\right]$. The RMAE becomes

$$\mathrm{RMAE}(\hat{\mathbf{P}}, \mathbf{P}^*) = \frac{\mathrm{MAE}(\hat{\mathbf{P}}, \mathbb{E}\left[\mathbf{X}\right])}{\mathbb{E}\left[\mathrm{MAE}(\hat{\mathbf{P}}, \mathbf{X})\right]}\,. \tag{4}$$

The MAE in Eq.(2) is a double sum of absolute values, which are convex functions, and the MAE is therefore convex. Jensen's inequality thus implies

$$\mathrm{RMAE}(\hat{\mathbf{P}}, \mathbf{P}^*) = \frac{\mathrm{MAE}(\hat{\mathbf{P}}, \mathbb{E}\left[\mathbf{X}\right])}{\mathbb{E}\left[\mathrm{MAE}(\hat{\mathbf{P}}, \mathbf{X})\right]} \leq \frac{\mathbb{E}\left[\mathrm{MAE}(\hat{\mathbf{P}}, \mathbf{X})\right]}{\mathbb{E}\left[\mathrm{MAE}(\hat{\mathbf{P}}, \mathbf{X})\right]} = 1\,. \tag{5}$$

Moreover, the MAE is obviously greater than or equal to 0 and since $\hat{\mathbf{P}}_{i,t} \in (0,1)$ by assumption, $|\hat{\mathbf{P}}_{i,t} - x_{i,t}| \in (0,1)$, meaning that $\mathbb{E}\left[\mathrm{MAE}(\hat{\mathbf{P}}, \mathbf{X})\right] > 0$ and $\mathrm{RMAE}(\hat{\mathbf{P}}, \mathbf{P}^*) \geq 0$. □

Like $U(\mathbf{X}\,|\,G)$, the case $\mathrm{RMAE}(\hat{\mathbf{P}}, \mathbf{P}^*) = 0$ occurs when all the elements of $\hat{\mathbf{P}}$ and $\mathbf{P}^*$ are equal—the conditional and marginal models are the same—and the case $\mathrm{RMAE}(\hat{\mathbf{P}}, \mathbf{P}^*) = 1$ occurs when $\mathbf{P}^* \in \{0,1\}^{N\times T}$—there is no uncertainty over $\mathbf{X}$ given G—and $\mathbf{P}^* \neq \hat{\mathbf{P}}$.

In Fig. 1, we compare the performance measures previously described with the uncertainty coefficients that helped us predict the existence of the coupling-duality in the context of the Glauber dynamics on small Erdős-Rényi graphs. We observe that the duality region between the prediction and reconstruction performance measures is analoguous to the one predicted by the uncertainty coefficients.

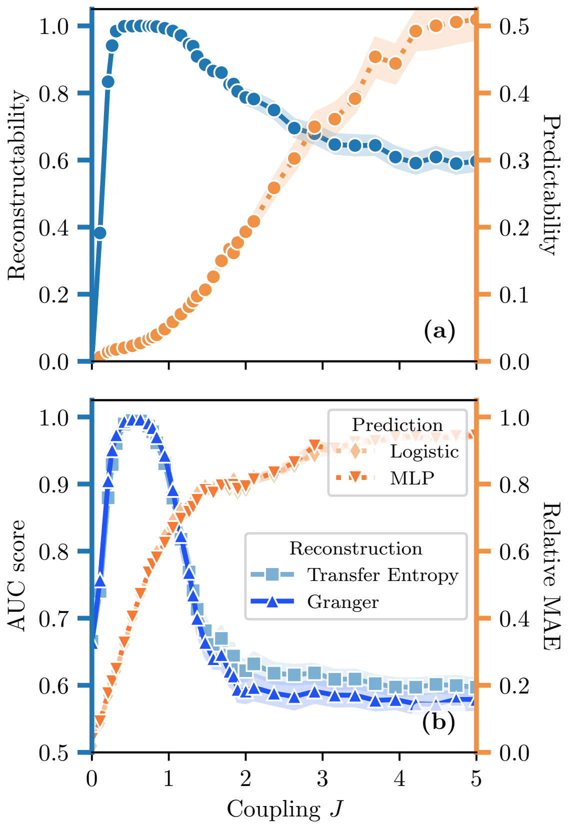

Supplementary Figure 1. **Coupling-duality versus prediction and reconstruction performance measures in the Glauber dynamics**: (a) coupling-duality between $U(G\,|\,\mathbf{X})$ (left axis) and $U(\mathbf{X}\,|\,G)$ (right axis), (b) duality between reconstruction AUC score (left axis) and prediction relative mean absolute error (right axis) for different algorithms as indicated by the legend. We fixed the number of nodes to $N = 5$, the number of edges to $M = 5$ and the number of time steps to $T = 100$, and we averaged each point over 1000 simulations. See Section IV.B for further detail about the performance measures and algorithms.

Supplementary Note II : Analytical solutions for simple systems

A. Two graphs and three time series

We consider the scenario presented in Section II.C of the main paper represented by Fig. 3(a). In this system, the structure can either be given by g_1 with probability $P(G = g_1) = p$ or g_2, with probability $P(G = g_2) = 1 - p$. For the purpose of the demonstration, the specifics of g_1 and g_2 are not needed. Then, g_1 can generate two time series, $\mathbf{x}_1$ and $\mathbf{X}_2$, with probabilities $P(\mathbf{X} = \mathbf{x}_1|G = g_1) = r$ and $P(\mathbf{X} = \mathbf{x}_2|G = g_1) = 1 - r$. Likewise, g_2 generates $\mathbf{x}_2$ with probability $P(\mathbf{X} = \mathbf{x}_2|G = g_2)$ and $\mathbf{x}_3$ with probability $P(\mathbf{X} = \mathbf{x}_3|G = g_2)$. In this example, it is assumed that $\mathbf{x}_1$, $\mathbf{x}_2$ and $\mathbf{x}_3$ are completely different time series.

First, we need to compute the mutual information $I(\mathbf{X}; G) = H(\mathbf{X}) - H(\mathbf{X} \mid G) = H(G) - H(G \mid \mathbf{X})$ in order to determine both uncertainty coefficients. For that, we first evaluate $H(\mathbf{X})$ and $H(\mathbf{X}|G)$:

$$\begin{aligned} H(\mathbf{X}) = &-pr\log(pr) \\ &- \Big[p(1-r) + (1-p)s\Big] \log\Big(p(1-r) + (1-p)s\Big) \\ &- (1-p)(1-s)\log\Big((1-p)(1-s)\Big) \end{aligned} \tag{6}$$

and

$$H(\mathbf{X}|G) = p\,\mathcal{H}(r) + (1-p)\,\mathcal{H}(s)\,, \tag{7}$$

such that $\mathcal{H}(q) = -q\log q - (1-q)\log(1-q)$ is the binary entropy. The mutual information is therefore the difference between the above equations.

$$\begin{aligned} I(\mathbf{X}; G) = &\; p(1-r)\log(1-r) + (1-p)s\log(s) \\ &- pr\log(p) - (1-p)(1-s)\log(1-p) \\ &- \Big[p(1-r) + (1-p)s\Big] \log\Big(p(1-r) + (1-p)s\Big) \end{aligned} \tag{8}$$

Then, we evaluate the entropy of the graph, which is simply given by $H(G) = \mathcal{H}(p)$.

Now, assuming that $s = 0$ gives us $I(\mathbf{X}; G) = \mathcal{H}(p)$, as mentioned in Section II.B, which yields $U(G \mid \mathbf{X}) = 1$. Also, since $H(\mathbf{X}) = p\mathcal{H}(r) + \mathcal{H}(p)$, then $U(\mathbf{X} \mid G) = \frac{\mathcal{H}(p)}{p\mathcal{H}(r)+\mathcal{H}(p)}$. When $s = 1$, we have $H(\mathbf{X}) = \mathcal{H}(pr)$ and $H(\mathbf{X}|G) = p\mathcal{H}(r)$, which leads to $I(\mathbf{X}; G) = \mathcal{H}(pr) - p\mathcal{H}(r)$. The uncertainty coefficients are, in this case, $U(\mathbf{X} \mid G) = 1 - \frac{p\mathcal{H}(r)}{\mathcal{H}(pr)}$ and $U(G \mid \mathbf{X}) = \frac{\mathcal{H}(pr)-p\mathcal{H}(r)}{\mathcal{H}(p)}$.

B. Markov process on a single node graph

Suppose the random graph G has only two instances: a single isolated node denoted as $a = 0$ with a probability of $(1 - p)$, and a single node connected to itself by a self-loop denoted as $a = 1$ with a probability of p. Thus, we have $H(G) = -p \log(p) - (1 - p) \log(1 - p)$. The state X_t of the node at time t is a Bernoulli random variable that evolves according to the following Markovian rule: if $X_{t-1} = 1$, then $X_t = 0$ with probability 1; if $X_{t-1} = 0$, then $X_t = 0$ with probability $1 - aq$, and $X_t = 1$ with probability aq. The initial condition remain fixed to $X_1 = 1$.

The parameter $q \in [0, 1]$ serves as a coupling between the structure and the function of the model. When $q = 0$, both graphs generate the same time series $(1, 0, 0, \ldots, 0)$. Therefore, observing the time series when $q = 0$ provides no information about the graph, resulting in minimal reconstructability $U(G \,|\, \mathbf{X}) = 0$. On the contrary, when $q = 1$, the graph with no self-loop can only generate the time series $(1, 0, 0, \ldots, 0)$ (absorbing state), while the graph with a self-loop can only generate the oscillating pattern $(1, 0, 1, 0, 1, \ldots)$. Thus, when $q = 1$ and $T > 2$, maximal reconstructability $U(G \,|\, \mathbf{X}) = 1$ is achieved: observing only one time series allows for the unique determination of the graph. Additionally, in this case, there is also maximal predictability $U(\mathbf{X} \,|\, G) = 1$ for $T > 1$, since observing the graph and the initial state 1 provides all subsequent values for X_t.

However, in general, when $0 < q < 1$, both reconstructability and predictability are only partial. Without a self-loop, there is still only one possible time series, namely $(1, 0, 0, \ldots, 0)$. With a self-loop, the aforementioned time series is possible, along with others such as $(1, 0, 1, 0, \ldots)$, $(1, 0, 0, 1, \ldots)$, and so on, with the restriction that state 1 cannot be followed by another state 1. Thus, because the time series $(1, 0, 0, \ldots, 0)$ has a non-zero probability of occurring in both graphs, the reconstructability satisfies $U(G \,|\, \mathbf{X}) < 1$. However, since the likelihood of $(1, 0, 0, \ldots, 0)$ decreases exponentially with T for the node with a self-loop, we know that $U(G \,|\, \mathbf{X}) \to 1$ as $T \to \infty$. Consequently, the mutual information approaches its maximal value $H(G)$ as $T \to \infty$. Similarly to the previous examples, this implies that the predictability behaves *dually* to the reconstructability, satisfying $U(\mathbf{X} \,|\, G) \to 0$ as $T \to \infty$.

Supplementary Note III: Predictability and reconstructability of deterministic dynamics

Our framework can be applied to continuous-state processes, where $x_i^*(t;g) \in \mathbb{R}$ is the solution of the process corresponding to the i-th node's state at continuous time $t \in [0,T]$ for the graph instance g of N nodes. For a large class of these processes, the states are governed by differential equations of the form

$$\frac{d}{dt}x_i^*(t;g) = F_1\Big(x_i^*(t;g)\Big) + \sum_{j=1}^{N} A_{ij}(g) F_2\Big(x_i^*(t;g), x_j^*(t;g)\Big)\,, \qquad i \in \{1,\dots,N\}\,, \tag{9}$$

where F_1 describes the self-dynamics of the nodes and F_2 specifies the interaction between connected nodes. A complete trajectory $\mathbf{x}^*(g)$ is given by a set of tuples $\boldsymbol{x}^*(t;g) = \big(x_1^*(t;g), \cdots, x_N^*(t;g)\big)$ for t in the interval $[0,T]$. Also, note that $A_{ij}(g)$ denotes the element (i,j) of the adjacency matrix of g, which equals one if there is an edge between i and j in graph g, and zero otherwise. The quench mean-field SIS dynamics can be formulated in this form, where $F_1(x) = \delta x$ and $F_2(x,y) = \lambda(1-x)y$. Other examples of F_1 and F_2 are provided in Refs. [1–4].

This class of system was investigated in Ref. [3], where it was found that many graphs can often generate similar time series. This conclusion suggests that predicting the evolution of a time series may in fact be possible without specific knowledge of the structure of interactions. Here, we shed light on this result using our information-theoretic framework.

To connect our framework with that of Ref. [3], we work in discrete time, where $t \in \big\{0, \Delta t, 2\Delta t, \cdots, (K-1)\Delta t\big\}$ for some small Δt. By doing so, the functions $x_i^*(t;g)$ are replaced by the matrix elements $x_{i,k}^*(g)$, where time steps are specified by the index $k \in \{0, 1, \dots, K-1\}$ as $t_k = k\Delta t$ and $T = t_{K-1} = (K-1)\Delta t$. The evolution equation (9) is then discretized as follows:

$$x_{i,k+1}^*(g) = x_{i,k}^*(g) + \Delta t \left[F_1\Big(x_{i,k}^*(g)\Big) + \sum_{j=1}^{N} A_{ij}(g) F_2\Big(x_{i,k}^*(g), x_{j,k}^*(g)\Big) \right] . \tag{10}$$

These processes are deterministic, meaning that for a fixed graph g and initial condition $\boldsymbol{x}_0^*(g) \in \mathbb{R}^N$, the probability that the trajectory $\boldsymbol{x}^*(g) \in \mathbb{R}^{N\times K}$ is sampled is, by definition, exactly equal to 1. We let the initial conditions be randomly chosen in a—discrete—set of states $\mathcal{X}_0 \subset \mathbb{R}^N$ with probability distribution $P(\boldsymbol{X}_0)$. Each trajectory generated by a graph g therefore becomes a $N \times K$ random matrix X whose values belong to a discrete set

$$\mathcal{X}(g) = \big\{x^*(g) \,:\, x_0^*(g) \in \mathcal{X}_0\big\}, \tag{11}$$

where we have omitted the explicit dependence of $\mathcal{X}(g)$ on $\mathcal{X}_0$ for brevity. The probability that the trajectory x is generated by g is

$$P\Big(X = x \Big| G = g\Big) = \begin{cases} P(\boldsymbol{X}_0 = \boldsymbol{X}_0) & \text{if } x \in \mathcal{X}(g)\,, \\ 0 & \text{otherwise}\,. \end{cases} \tag{12}$$

This is consistent with the equality $|\mathcal{X}(g)| = |\mathcal{X}_0|$, which is a direct consequence of the deterministic nature of the dynamics. Hence, all the stochasticity of the system is coming from the choice of initial conditions. For the remainder of the calculation, we will assume for simplicity that the initial conditions are uniformly distributed, i.e., $P(\boldsymbol{X}_0 = \boldsymbol{x}_0) = |\mathcal{X}_0|^{-1}$. This requires $\mathcal{X}_0$ to be finite so that the set of all trajectories generated by all graphs of N nodes in some finite set $\mathcal{G}$,

$$\mathcal{X} = \big\{\mathbf{x} \in \mathcal{X}(g) \,:\, g \in \mathcal{G}\big\}, \tag{13}$$

also remains finite. This technicality allows the entropies of $\mathbf{X}$ to remain finite and well defined, something necessary to keep our framework unchanged. Note that the case where $\mathcal{X}_0$ is countably infinite can also be treated by properly choosing a non-uniform distribution $P(\boldsymbol{X}_0)$. Further work is required if a truly continuous-state formulation of our information measures is to be considered.

This description of a deterministic system is quite restrictive, since by construction a graph is only allowed to generate a single time series once the initial conditions have been fixed. We would like to relax the notion of deterministic systems to admit a prediction error ϵ. Let

$$\mathcal{X}_\epsilon(g) = \big\{\mathbf{x} \in \mathcal{X} \,:\, \|\mathbf{x} - \mathbf{x}^*(g)\| \leq \epsilon,\, \mathbf{x}^*(g) \in \mathcal{X}(g)\big\}, \tag{14}$$

be the set of all trajectories $\mathbf{x}$ that are sufficiently close—up to a distance $\epsilon \geq 0$—to a trajectory $\mathbf{x}^*(g)$ deterministically generated by g, where the symbol $\|\cdot\|$ denotes some matrix norm. We say that a trajectory $\mathbf{x}$ is ϵ-compatible with g if $\mathbf{x} \in \mathcal{X}_\epsilon(g)$. In other words, $\mathbf{x}$ is ϵ-compatible with g if it is deterministically generated by g or if there is another graph $h \in \mathcal{G}$ that deterministically generate a trajectory $\mathbf{x}^*(h)$ that satisfies $\mathbf{x}^*(h) = \mathbf{x}$ and $\|\mathbf{x}^*(h) - \mathbf{x}^*(g)\| \leq \epsilon$. Likewise, we define $\mathcal{G}_\epsilon(\mathbf{x}) = \{g \in \mathcal{G} \,:\, \mathbf{x} \in \mathcal{X}_\epsilon(g)\}$, i.e., the set of all graphs g that are ϵ-compatible with $\mathbf{x}$. Thus, $\mathbf{x}$ is ϵ-compatible with g iff g is ϵ-compatible with $\mathbf{x}$. In this scenario, we introduce a new $N \times K$ random matrix $\mathbf{X}_\epsilon$, taking values in the set of all deterministic trajectories $\mathcal{X}$, defined in Eq. (13). The probability of $\mathbf{X}_\epsilon = \mathbf{x}$ given $G = g$, interpreted as the probability that the trajectory $\mathbf{x}$ is generated from g within a margin of error of size ϵ, is simply defined as the uniform distribution over all trajectories in $\mathcal{X}_\epsilon(g)$:

$$P\Big(\mathbf{X}_\epsilon = \mathbf{x} \Big| G = g\Big) = \begin{cases} |\mathcal{X}_\epsilon(g)|^{-1} & \text{if } \mathbf{x} \in \mathcal{X}_\epsilon(g), \\ 0 & \text{otherwise.} \end{cases} \tag{15}$$

As we approach the limit $\epsilon \to 0^+$, the normalization factor $|\mathcal{X}_\epsilon(g)|$ tends to $|\mathcal{X}(g)| = |\mathcal{X}_0|$ for all $g \in \mathcal{G}$, and the above conditional probability converges towards Eq. (12) defining $\mathbf{X}$ (with uniformly distributed initial conditions). Moreover, for ϵ sufficiently large, all conditional probabilities become non-zero. Increasing ϵ therefore introduces uncertainty about which graph can generate a specific trajectory, gradually blurring the deterministic nature of the process.

At this point, we have all the tools to compute the predictability and reconstructability. We begin our calculation with the mutual information. First, we evaluate the marginal probability of $\mathbf{X}_\epsilon$ as follows:

$$P(\mathbf{X}_\epsilon = \mathbf{x}) = \sum_{g\in\mathcal{G}} P(G=g)P(\mathbf{X}_\epsilon = \mathbf{x}|G=g) = \sum_{g\in\mathcal{G}_\epsilon(\mathbf{x})} \frac{p(g)}{|\mathcal{X}_\epsilon(g)|},$$

where $p(g)$ denotes the probability $P(G=g)$. From there, we calculate the entropies and the mutual information:

$$H(\mathbf{X}_\epsilon|G) = \mathbb{E}_G\left[\log|\mathcal{X}_\epsilon(G)|\right], \tag{16a}$$

$$H(\mathbf{X}_\epsilon) = -\mathbb{E}_{\mathbf{X}_\epsilon}\left[\log\left(\sum_{h\in\mathcal{G}_\epsilon(\mathbf{X}_\epsilon)} \frac{p(h)}{|\mathcal{X}_\epsilon(h)|}\right)\right], \tag{16b}$$

$$I(\mathbf{X}_\epsilon;G) = -\mathbb{E}_{G,\mathbf{X}_\epsilon}\left[\log\left(\sum_{h\in\mathcal{G}_\epsilon(\mathbf{X}_\epsilon)} p(h)\frac{|\mathcal{X}_\epsilon(G)|}{|\mathcal{X}_\epsilon(h)|}\right)\right], \tag{16c}$$

Using this expression for the mutual information leads to the following predictability:

$$U(\mathbf{X}_\epsilon|G) = \frac{I(\mathbf{X}_\epsilon;G)}{H(\mathbf{X}_\epsilon)} = \frac{\mathbb{E}_{G,\mathbf{X}_\epsilon}\left[\log\left(\sum_{g\in\mathcal{G}_\epsilon(\mathbf{X}_\epsilon)} p(g)\frac{|\mathcal{X}_\epsilon(G)|}{|\mathcal{X}_\epsilon(g)|}\right)\right]}{\mathbb{E}_{\mathbf{X}_\epsilon}\left[\log\left(\sum_{g\in\mathcal{G}_\epsilon(\mathbf{X}_\epsilon)} \frac{p(g)}{|\mathcal{X}_\epsilon(g)|}\right)\right]} \tag{17}$$

$$= 1 - \frac{H(\mathbf{X}_\epsilon|G)}{H(\mathbf{X}_\epsilon)} = 1 - \frac{\mathbb{E}_G\left[\log|\mathcal{X}_\epsilon(G)|\right]}{\mathbb{E}_{\mathbf{X}_\epsilon}\left[\log\left(\sum_{g\in\mathcal{G}_\epsilon(\mathbf{X}_\epsilon)} \frac{p(g)}{|\mathcal{X}_\epsilon(g)|}\right)^{-1}\right]} \tag{18}$$

Also, since $H(G) = -\mathbb{E}_G\left[\log P(G)\right]$, the reconstructability is given by

$$U(G|\mathbf{X}_\epsilon) = \frac{I(\mathbf{X}_\epsilon;G)}{H(G)} = \frac{\mathbb{E}_{G,\mathbf{X}_\epsilon}\left[\log\left(\sum_{h\in\mathcal{G}_\epsilon(\mathbf{X}_\epsilon)} p(h)\frac{|\mathcal{X}_\epsilon(G)|}{|\mathcal{X}_\epsilon(h)|}\right)\right]}{\mathbb{E}_G\left[\log P(G)\right]} \tag{19}$$

$$= 1 - \frac{H(G|\mathbf{X}_\epsilon)}{H(G)} = 1 - \frac{\mathbb{E}_{G,\mathbf{X}_\epsilon}\left[\log\left(\sum_{h\in\mathcal{G}_\epsilon(\mathbf{X}_\epsilon)} \frac{p(h)}{P(G)}\frac{|\mathcal{X}_\epsilon(G)|}{|\mathcal{X}_\epsilon(h)|}\right)\right]}{\mathbb{E}_G\left[\log P(G)\right]}. \tag{20}$$

To build more intuition about our theoretical results, let us focus on the predictability and consider two opposite limit cases: $\epsilon \to 0^+$ (minimum error tolerance) and $\epsilon \to \infty$ (maximum error tolerance). As ϵ approaches these two limits, $|\mathcal{X}_\epsilon(g)|$ respectively tends to $|\mathcal{X}_0|$ (number of

initial conditions) and $|\mathcal{X}|$ (number of trajectories generated by all possible graphs from all initial conditions). This results hold for all $g \in \mathcal{G}$. Hence,

$$H(\mathbf{X}_\epsilon|G) \to \begin{cases} \log|\mathcal{X}_0| & \epsilon \to 0^+, \\ \log|\mathcal{X}| & \epsilon \to \infty\,. \end{cases} \tag{21}$$

Moreover,

$$H(\mathbf{X}_\epsilon) \to \begin{cases} \log|\mathcal{X}_0| + \mathcal{I} & \epsilon \to 0^+, \\ \log|\mathcal{X}| & \epsilon \to \infty\,. \end{cases} \tag{22}$$

where $\mathcal{I} = -\mathbb{E}_{\mathbf{X}}\left[\log\sum_{g\in\mathcal{G}(\mathbf{X})} p(g)\right] > 0$, with $\mathcal{G}(\mathbf{x}) = \mathcal{G}_0(\mathbf{x})$ being the set of all graphs that deterministically generate the trajectory $\mathbf{x}$ starting from some initial condition in $\mathcal{X}_0$. Note that $\mathcal{I}$ is the limit of the mutual information as $\epsilon \to 0^+$ and can be written as $\mathbb{E}_{\mathbf{X}}\left[\log q(\mathbf{X})^{-1}\right]$, where

$$q(\mathbf{x}) = \sum_{g\in\mathcal{G}(\mathbf{x})} p(g) \tag{23}$$

is the probability to have a graph in $\mathcal{G}(\mathbf{x})$. Going back to the expression for the predictability, we conclude that

$$U(\mathbf{X}_\epsilon|G) \to \begin{cases} 1 - \frac{\log|\mathcal{X}_0|}{\log|\mathcal{X}_0|+\mathcal{I}} & \epsilon \to 0^+, \\ 0 & \epsilon \to \infty\,. \end{cases} \tag{24}$$

The last result is easy to interpret. On the one hand, when no margin of error is allowed and the graph is known, the only factor limiting our knowledge about the states' evolution is the uncertainty about the initial conditions. Once an initial condition is chosen, corresponding to $|\mathcal{X}_0| = 1$, no uncertainty remains and the predictability reaches its maximal value 1, thus aligning with the intuition that in a deterministic process as in Eq. (10), the initial condition and the graph entirely determine the future. On the other hand, when all errors are tolerated between different trajectories, a single graph is seen as capable of generating all trajectories. Consequently, knowing G provides no information about the states that will follow the initial conditions, in perfect accordance with a zero predictability. Interpolating between these extreme cases, we understand that as ϵ decreases, the number of trajectories that are ϵ-compatible with a specific graph also decreases, meaning that on average, the uncertainty about the evolution of the process that remains after the observation of a graph also decreases, resulting in an increase of predictability.

Even substantial values of ϵ can lead to high values of predictability. To understand this last claim, let us rewrite the entropy of the process as

$$H(\mathbf{X}_\epsilon) = \mathcal{A}_\epsilon + H(G) - \mathbb{E}_{G,\mathbf{X}_\epsilon}\left[\log R_\epsilon(G,\mathbf{X}_\epsilon)\right], \tag{25}$$

where

$$R_\epsilon(g,\mathbf{x}) = \sum_{h\in\mathcal{G}_\epsilon(\mathbf{x})} \frac{p(h)}{p(g)}\frac{|\mathcal{X}_\epsilon(g)|}{|\mathcal{X}_\epsilon(h)|}\,, \quad \mathcal{A}_\epsilon = \mathbb{E}_G\left[\log|\mathcal{X}_\epsilon(G)|\right] \tag{26}$$

Then, assuming that the graphs are almost uniformly distributed (all graphs sharing the same a priori contribution to the process) and possesses almost the same number of ϵ-compatible trajectories, we find that

$$R_\epsilon(g,\mathbf{x}) \approx \sum_{h\in\mathcal{G}_\epsilon(\mathbf{x})} 1 = |\mathcal{G}_\epsilon(\mathbf{x})| \tag{27}$$

for all graphs g. Moreover, in such circumstances, $H(G)\approx \log|\mathcal{G}|$ and this leads to an approximate formula for the mutual information:

$$I(\mathbf{X}_\epsilon;G) \approx \mathcal{I}_\epsilon = \mathbb{E}_{\mathbf{X}_\epsilon}\left[\log\frac{|\mathcal{G}|}{|\mathcal{G}_\epsilon(\mathbf{X}_\epsilon)|}\right]\,, \tag{28}$$

which in turn leads to an approximate formula for the predictability:

$$U(\mathbf{X}_\epsilon|G) \approx 1 - \frac{\mathcal{A}_\epsilon}{\mathcal{A}_\epsilon + \mathcal{I}_\epsilon}\,. \tag{29}$$

Therefore, provided $|\mathcal{G}_\epsilon(\mathbf{x})|$ remains substantially smaller than $|\mathcal{G}|$ for every $\mathbf{x}$, a high level of predictability is retained. *This means that even in the presence of numerous graphs capable of producing a trajectory within an error margin of ϵ (though considerably fewer relative to the total graph count), high predictability is feasible. This observation reinforces the notion that an exclusive relationship between a graph and a time series isn't a prerequisite for high predictability, echoing the conclusions drawn in Ref. [3].*

We now analyze the limit cases of the reconstructability given in Eqs. (19)–(20). Since the graph distribution $P(G)$ is not affected by the error margin ϵ, leaving the corresponding entropy $H(G)$ unchanged, the only factor that influences the reconstructability is the mutual information in Eq. (16c). Considering $\epsilon\to 0^+,\infty$ allows us to simplify its expression quite significantly as

$$I(\mathbf{X}_\epsilon;G) \to \begin{cases} \mathcal{I} & \epsilon\to 0^+, \\ 0 & \epsilon\to\infty, \end{cases} \tag{30}$$

where we recall that $\mathcal{I} = \mathbb{E}_{\mathbf{X}}\left[\log q(\mathbf{X})^{-1}\right] > 0$ with $q(\mathbf{x})$ defined by Eq. (23). This naturally leads to the following reconstructability:

$$U(G|\mathbf{X}_\epsilon) \to \begin{cases} 1 - \frac{H(G)-\mathcal{I}}{H(G)} & \epsilon\to 0^+, \\ 0 & \epsilon\to\infty. \end{cases} \tag{31}$$

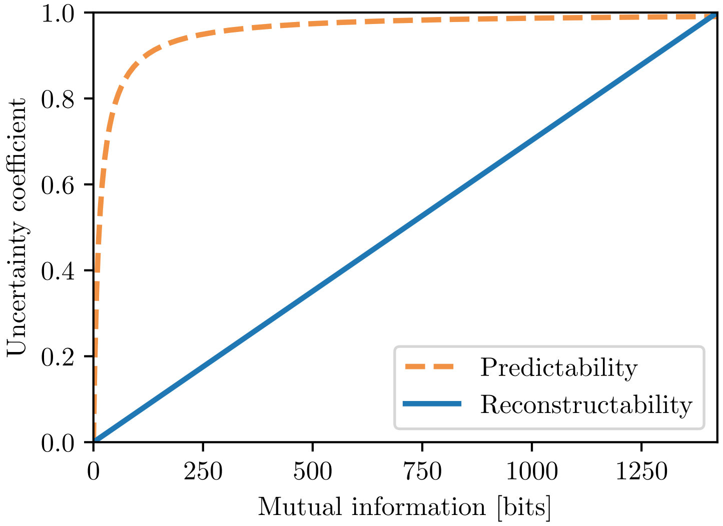

Supplementary Figure 2. Scaling of the uncertainty coefficients with the mutual information. We show the approximations of $U(\mathbf{X}_\epsilon|G)$ and $U(G|\mathbf{X}_\epsilon)$, provided by Eqs (31) and (33), respectively. For this illustration, we fixed $\log|\mathcal{G}| = \log\binom{N(N-1)/2}{M}$ and $\mathcal{A}_\epsilon = \log(\alpha|\mathcal{X}_0|)$, with $N = 100$, $M = 250$ and $\alpha = |\mathcal{X}_0| = 100$. While the choice of $\log|\mathcal{G}|$ is easy to justify—we assume that $\mathcal{G}$ is the set of all graphs with $N = 100$ nodes and $M = 250$ edges—, the choice for $\mathcal{A}_\epsilon$ needs more explanation. It can be interpreted as a system where, for each initial condition, there are on average α trajectories that are in the neighborhood of a given trajectory deterministically generated by some graph g. Here, we assume that α and $|\mathcal{X}_0|$ are equal to N for simplicity.

Again, the interpretation of this reconstructability is simple. First, with maximal tolerance for error, any graph is ϵ-compatible with every other trajectories. Consequently, knowing $\mathbf{X}$ does not provide information about G whatsoever and the reconstructability is zero. Second, when we gradually decrease the margin of error, fewer graphs are ϵ-compatible with any trajectory, providing more and more information about G. Hence, like $U(\mathbf{X}_\epsilon|G)$, $U(G|\mathbf{X}_\epsilon)$ increases with decreasing error tolerance size ϵ. Reconstructability is maximized when $\epsilon \to 0^+$, in which case its formula is easier to interpret when G is uniformly distributed, i.e., $P(G) = |\mathcal{G}|^{-1}$. In this case, $q(\mathbf{x}) = \frac{|\mathcal{G}(\mathbf{x})|}{|\mathcal{G}|}$ and $I(\mathbf{X};G) = \log|\mathcal{G}| - \mathbb{E}_\mathbf{X}\left[\log|\mathcal{G}(\mathbf{X})|\right]$, leading to

$$\lim_{\epsilon\to 0^+} U(G|\mathbf{X}_\epsilon) = 1 - \frac{\mathbb{E}_\mathbf{X}\left[\log|\mathcal{G}(\mathbf{X})|\right]}{\log|\mathcal{G}|}. \tag{32}$$

When no margin of error is tolerated, the reconstructability of G given $\mathbf{X}$ is simply given by the ratio between the logarithms of the number of graphs that are compatible with any given trajectories (on average), and the total number of graphs. As fewer graphs become on average compatible with any trajectory, the reconstructability converges to 1. In other words, reconstructability boils down

to the fraction of graphs that may have generated $\mathbf{X}$: the more compatible graphs there are, the harder it gets to find the correct graph.

Using similar assumptions as before, we can evaluate an approximate formula for the reconstructability as well. Again, assuming that $P(G)$ is almost uniform and ϵ is sufficiently small so that Eq. (30) is valid leads to

$$U(G|\mathbf{X}_\epsilon) \approx 1 - \frac{\log|\mathcal{G}| - \mathcal{I}_\epsilon}{\log|\mathcal{G}|}\,. \tag{33}$$

Using our framework allows us to reach similar conclusions to the ones in Ref. [3] described above. Indeed, we find that, for sufficiently small tolerance ϵ, it is possible to have poor reconstructability even though the predictability is almost equal to one (see Fig. 2). This is because of the different scaling behaviors with respect to the mutual information of $U(\mathbf{X}_\epsilon|G)$ and $U(G|\mathbf{X}_\epsilon)$: $U(\mathbf{X}_\epsilon|G)$ quickly saturates to one when I_ϵ increases, whereas $U(G|\mathbf{X}_\epsilon)$ grows linearly with I_ϵ. While we corroborate the findings of Ref. [3], it is important to stress that our results are conceptually quite different. For instance, their notion of reconstructability is intrinsically related to which graphs are reconstructed by their algorithm, and how different they are from the original graph. In turn, they measure reconstructability using the AUC score of the reconstructed graph which does not incorporate the full range of graphs that can generate each specific trajectory. In this respect, our framework offers a different and complementary perspective to their work, by quantifying their observation in terms of information.

Supplementary Note IV : Proof of the θ-duality between extrema

In this section, we relate the presence of extrema of $U(\mathbf{X}\,|\,G)$ and $U(G\,|\,\mathbf{X})$ with the existence of a θ-duality.

Lemma 2 (θ-duality between extrema)**.** *Let Θ be a non-empty subinterval of the variable θ whose one endpoint is a local extremum of $U(\mathbf{X}\,|\,G)$ and the other, a local extremum of $U(G\,|\,\mathbf{X})$. Moreover, suppose that $U(\mathbf{X}\,|\,G)$ and $U(G\,|\,\mathbf{X})$ do not have critical points in Θ. Then the extrema points delineate a region of θ-duality if and only if they are both maxima (or both minima).*

Proof. Let θ_R and θ_P be the extrema points of $U(G\,|\,\mathbf{X})$ and $U(\mathbf{X}\,|\,G)$, respectively. Thus

$$\left.\frac{\partial U(G\,|\,\mathbf{X})}{\partial\theta}\right|_{\theta=\theta_R} = \left.\frac{\partial U(\mathbf{X}\,|\,G)}{\partial\theta}\right|_{\theta=\theta_P} = 0\,. \tag{34}$$

Suppose for a moment that $\theta_R < \theta_P$ and let $\Theta = (\theta_R, \theta_P)$. This implies that $\frac{\partial U(G\,|\,\mathbf{X})}{\partial\theta}$ changes sign at θ_R, before $\frac{\partial U(\mathbf{X}\,|\,G)}{\partial\theta}$, for which the sign change happens at θ_P.

On the one hand, if the extrema points θ_R and θ_P are both maxima (or minima), then $\frac{\partial U(G\,|\,\mathbf{X})}{\partial\theta}$ and $\frac{\partial U(\mathbf{X}\,|\,G)}{\partial\theta}$ have different signs in Θ. Hence, the inequality

$$\left[\frac{\partial U(\mathbf{X}\,|\,G)}{\partial\theta}\frac{\partial U(G\,|\,\mathbf{X})}{\partial\theta}\right]_{\theta=\theta^*} < 0 \tag{35}$$

is verified in this region. The uncertainty coefficients are therefore θ-dual in Θ.

On the other hand, if the uncertainty coefficients are θ-dual in Θ, then inequality (35) is satisfied in this interval. This in turn implies that either $U(G\,|\,\mathbf{X})$ decreases in Θ while $U(\mathbf{X}\,|\,G)$ increases or $U(G\,|\,\mathbf{X})$ increases in Θ while $U(\mathbf{X}\,|\,G)$ decreases. Therefore, the endpoints of Θ are either both maximum points or both minimum points.

Finally, repeating the same arguments with $\theta_R > \theta_P$ and $\Theta = (\theta_P, \theta_R)$ leads to the same conclusions about θ-duality of $U(\mathbf{X}\,|\,G)$ and $U(G\,|\,\mathbf{X})$ in Θ. □

Supplementary Note V: Proof of the monotonicity of $I(\mathbf{X}; G)$ with T

In this section, we prove the monotonicity of $I(\mathbf{X}; G)$ with respect to T for completing the proof of the universality of the T-duality discussed in the main paper, where T is the length of the process $\mathbf{X}$. The lemma is stated as follows:

Lemma 3 (Monotonicity of mutual information information with T)**.** *Let* $\mathbf{X} = (\boldsymbol{X}_1, \boldsymbol{X}_2, \cdots, \boldsymbol{X}_T)$ *be a Markov chain of length T whose transition probabilities are conditional to some discrete random variable G that is independent of T and such that* $H(\boldsymbol{X}_{t+1}|\boldsymbol{X}_t) > 0$ *for all* $t \in \{1, \ldots, T-1\}$. *Suppose moreover that the state spaces of $\mathbf{X}$ and G are finite. Then the mutual information $I(\mathbf{X}; G)$ is nonzero and monotonically increasing with $T \in \mathbb{Z}_+$.*

Proof. Let us define a Markov chain $\mathbf{X}' = (\boldsymbol{X}_1, \boldsymbol{X}_2, \cdots, \boldsymbol{X}_{T-1})$ of size $T-1$, such that the concatenation of $\mathbf{X}'$ with state variable $\boldsymbol{X}_T$ yields $\mathbf{X}$. Hence, we can express the mutual information between $\mathbf{X}$ and G in terms of $\mathbf{X}'$ as $I(\mathbf{X}; G) = I(\mathbf{X}', \boldsymbol{X}_T; G)$. Furthermore, proving the monotonicity of mutual information can be reformulated as proving the following inequality:

$$I(\mathbf{X}', \boldsymbol{X}_T; G) - I(\mathbf{X}'; G) > 0\,, \tag{36}$$

for all T. By the chain rule for conditional mutual information, that is $I(\mathbf{X}', \boldsymbol{X}_T; G) = I(\boldsymbol{X}_T; G|\mathbf{X}') + I(\mathbf{X}'; G)$, inequality (36) becomes

$$I(\boldsymbol{X}_T; G|\mathbf{X}') = H(\boldsymbol{X}_T|\mathbf{X}') - H(\boldsymbol{X}_T \mid \mathbf{X}', G) > 0\,. \tag{37}$$

The term $H(\boldsymbol{X}_T|\mathbf{X}') - H(\boldsymbol{X}_T \mid \mathbf{X}', G)$ is always at least non-negative, by virtue of the non-negativity of mutual information [5, Theorem 2.6.5]. Then, to prove inequality (37), we must verify that $H(\boldsymbol{X}_T|\mathbf{X}')$ never equals $H(\boldsymbol{X}_T \mid \mathbf{X}', G)$. Recalling that $H(\boldsymbol{X}_T \mid \mathbf{X}') \geq H(\boldsymbol{X}_T \mid \mathbf{X}', G) \geq 0$, inequality (37) does not hold if (i) $H(\boldsymbol{X}_T|\mathbf{X}') = 0$ or if (ii) $\boldsymbol{X}_T$ is independent of G (i.e., $I(\boldsymbol{X}_T; G|\mathbf{X}') = 0$). According to the hypothesis $H(\boldsymbol{X}_{t+1}|\boldsymbol{X}_t) > 0$ for all $t \in \{1, \ldots, T-1\}$, condition (i) cannot be true. Moreover, condition (ii) implies that $I(\mathbf{X}; G) = I(\boldsymbol{X}_T, \mathbf{X}'; G) = I(\mathbf{X}'; G) = 0$. Therefore, the only instance where Eq. (36) is not satisfied is when the Markov chain $\mathbf{X}$ is independent of G, i.e., $I(\mathbf{X}; G) = 0$ for all length T. However, this contradicts the assumption about the transition probabilities. Hence, $I(\mathbf{X}; G) > 0$ and monotonically increases with T. $\square$

We have to make a few remarks about the restrictions imposed in the last lemma. The condition $H(\boldsymbol{X}_{t+1}|\boldsymbol{X}_t) > 0$ for all $t \in \{1, \ldots, T-1\}$ only asserts that the Markov chain is nondeterministic

in the sense that knowing the state of the chain at time t does not completely eliminate the uncertainty about the state at time $t+1$. This condition is satisfied for wide variety of stochastic processes, including the irreducible Markov chains, where there is always a nonzero probability to transition from a state to any other state in a finite number of time steps.

Moreover, the finiteness of the state spaces for the chain $\mathbf{X}$ and the variable G is imposed to make $H(\mathbf{X})$, $H(G)$, and $I(\mathbf{X};G)$ finite. This in turn ensures that the uncertainty coefficients $U(G \mid \mathbf{X})$ and $U(\mathbf{X} \mid G)$ are well defined for all $T \in \mathbb{Z}_{+}$, a property that is necessary to prove the next lemma.

Supplementary Note VI: Proof of the existence of continuous extensions of uncertainty coefficients with T

In this section, we prove the existence of continuous extensions of the uncertainty coefficients of $I(\mathbf{X};G)$ with respect to T for completing the proof of the universality of the T-duality discussed in the main paper, where T is the length of the process $\mathbf{X}$. The lemma is stated as follows:

Lemma 4 (Continuous extension of uncertainty coefficients with T)**.** *Let* $\mathbf{X} = (\boldsymbol{X}_1, \boldsymbol{X}_2, \cdots, \boldsymbol{X}_T)$ *and* G *respectively be a Markov chain and a discrete random variable as in Lemma 3. Then the uncertainty coefficients* $U(G\,|\,\mathbf{X})$ *and* $U(\mathbf{X}\,|\,G)$*, interpreted as functions of* $T \in \mathbb{Z}_+$*, can be uniquely generalized to functions, respectively* $f(T)$ *and* $g(T)$*, that are holomorphic for all* $T \in \mathbb{C}$*, and thus real analytic for all* $T \in \mathbb{R}_+$*. Moreover,* $H(\mathbf{X})$ *can be extended to a function* $h(T)$ *that is analytic for all* $T \in \mathbb{R}_+$ *except where* $f(T) = 0$*.*

Proof. We first consider $U(\mathbf{X}\,|\,G)$ and $U(G\,|\,\mathbf{X})$. These can be interpreted as functions of $T \in \mathbb{Z}_+$ whose values belong to the interval $[0,1]$. According to Guichard's Theorem [6, Theorem 5.2.1] (see also [7, Theorem 15.13]), there exist two functions of $z \in \mathbb{C}$, denoted f and g, that are holomorphic in the whole complex plane and whose values at $z = T \in \mathbb{Z}_+$ equal those of $U(\mathbf{X}\,|\,G)$ and $U(G\,|\,\mathbf{X})$, respectively.

Now, $U(\mathbf{X}\,|\,G)$ and $U(G\,|\,\mathbf{X})$, and consequently $f(z)$ and $g(z)$, have bounded values for all $z = T \in \mathbb{Z}_+$. Moreover, f and g are holomorphic, so their restriction to the axis $z = T \in \mathbb{R}$ is real analytic. Hence, on that axis, f and g are Lipschitz continuous, which means that there are positive and finite constants, a and b, such that $|f(T) - f(T')| \leq a|T - T'|$ and $|g(T) - g(T')| \leq b|T - T'|$ for all $T, T' \in \mathbb{R}$. Choosing $T = T' + \epsilon$ with $T' \in \mathbb{Z}_+$ and $|\epsilon| < 1$, we conclude that $f(T)$ and $g(T)$ have finite values for all $T \in \mathbb{R}_+$.

The functions f and g are thus holomorphic in the whole complex plane and bounded on the positive real axis. This allows to use a special case of Carlson's Theorem [8, Theorem 2.8.1] according to which holomorphic functions that are bounded on the positive real axis are uniquely defined by their values on the set $\mathbb{Z}_+$. Therefore, f is the unique extension $U(\mathbf{X}\,|\,G)$ that is holomorphic for all $T \in \mathcal{C}$. Note that the restriction of f on the positive real axis is real analytic on this domain. Thus, there is a unique extension of $U(\mathbf{X}\,|\,G)$ that is real analytic for all $T \in \mathbb{R}_+$ and that can be further extended to a holomorphic function for all $T \in \mathbb{C}$. The same conclusion holds for g and $U(G\,|\,\mathbf{X})$.

To finish the proof, we need to tackle $H(\mathbf{X})$. We cannot use the same strategy as above because

$H(\mathbf{X})$ is not a bounded function of $T \in \mathbb{Z}_+$. However, by definition, the identity

$$H(\mathbf{X}) = \frac{H(G)U(G \,|\, \mathbf{X})}{U(\mathbf{X} \,|\, G)} \,. \tag{38}$$

is valid whenever $U(\mathbf{X} \,|\, G) > 0$. Now, according to Lemma 3, $I(\mathbf{X}; G) > 0$ and hence $U(\mathbf{X} \,|\, G) > 0$ for all $T \in \mathbb{Z}_+$. This means that Eq. (38) is well defined for all $T \in \mathbb{Z}_+$. To extend the domain of validity of the identity, we use the analytic functions f and g introduced above and define a new function h as

$$h(T) = H(G)\frac{g(T)}{f(T)} \,. \tag{39}$$

The values of h coincide with those of $H(\mathbf{X})$ for all $T \in \mathbb{Z}_+$, so that Eq. (38) defines a unique extension of $H(\mathbf{X})$. Moreover, h is analytic for all $T \in \mathbb{R}_+$ except at the points T where $f(T) = 0$. □

Lemma 4 ensures the *existence of analytic extensions* for the uncertainty coefficients, considered as functions of the positive integer T. These extensions can thus be evaluated and derived without restriction on the whole domain $\mathbb{R}_+$, which is a desirable property that is exploited in the proof of Theorem 1. However, the same lemma does not guarantee the monotonicity of the extensions on $\mathbb{R}_+$ in the event where they are monotone on $\mathbb{Z}_+$, although we assume it when proving Theorem 1. This is a reasonable assumption since numerical methods, generalizing the well-known Fritsch-Butland algorithm [9], have been recently developed to *construct smooth* (i.e., at least continuously differentiable) *and monotone interpolating functions* from any finite monotone datasets [10, 11]. With this assumption in hand, together with Lemmas 3 and 4, we can prove our main theoretical result (Theorem 1, Section II-D of the main paper): the universality of the T-duality in Markov chains. The proof is provided in Section III.D of the main paper.

Supplementary Note VII: Numerical analysis of the past-dependent measures

In this section, we investigate further the past-dependent measures presented in Section II.B. Specifically, we discuss the implication of the universality of the T-duality (Theorem 1, see main text) in a more general context, using the past-dependent mutual information $I(\mathbf{X}_{\text{future}}; G|\mathbf{X}_{\text{past}})$.

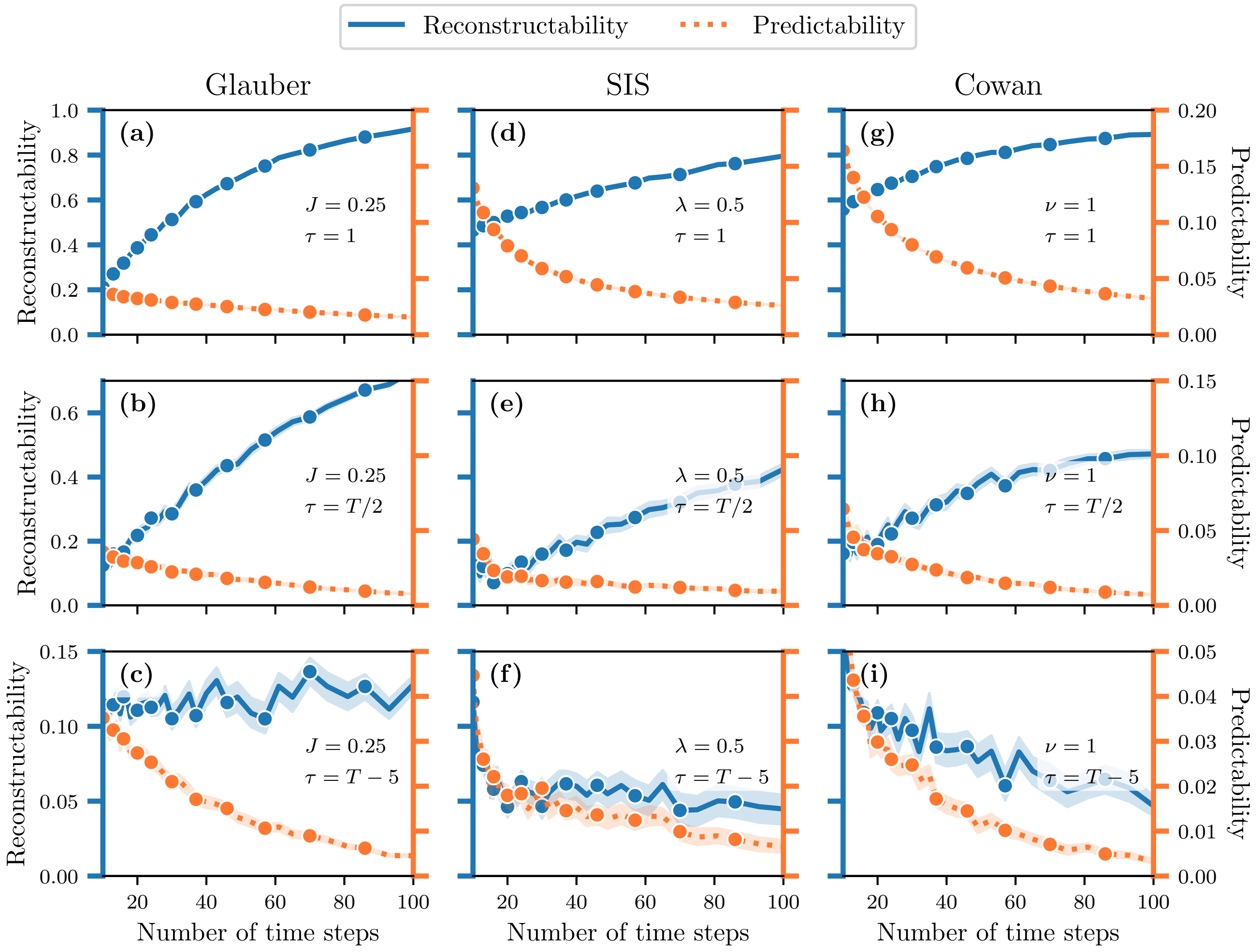

Supplementary Figure 3. Existence of the T-duality in the past-dependent case, for binary dynamics evolving on small Erdős-Rényi random graphs: (a-c) Glauber dynamics, (d-f) SIS dynamics and (g-i) Cowan dynamics. Like Fig 5 of the main paper, each panel shows the reconstructability coefficient $U(G\,|\,\mathbf{X}) \in [0,1]$ (blue) and the predictability coefficient $U(\mathbf{X}\,|\,G) \in [0,1]$ (orange) as a function of the number of time steps T. In each row, we change the value of the length τ of the past Markov chain $\mathbf{X}$: (a,d,g) $\tau = 1$, (b,e,h) $\tau = T/2$ and (c,f,i) $\tau = T - 5$. We used graphs of $N = 5$ vertices and $E = 5$ edges and each symbol corresponds to the average value measured over 1000 samples. We also show different values of the coupling parameters, as indicated on each figure.

We show in Fig. 3 the reconstructability and predictability for increasing process length T, and different values of τ.

Two scenarios are of interest: The case where τ is constant with respect to T and the case where it is not. When τ is constant with respect to T, Theorem I remains valid since the additional conditions on the Markov chain Y and the random graph G are a special case of the prior assumptions. Hence, we observe the T-duality for any value of τ in this case, as supported by Figs. 3(a,d,g).

The second scenario, when τ is a function of T, is more nuanced, as seen in Figs. 3(b-c,e-f,h-i) since Theorem 1 no longer applies. This is because both Y and G (represented by $\mathbf{X}$ and Y in Theorem 1, respectively) are now conditioned on $\mathbf{X}$, and thus will depend on T. Consequently, we no longer can assume that the entropy rate of Y given $\mathbf{X}$ is constant with T and that $H(G \mid \mathbf{X})$ is independent of T. In Fig. 3, we break this scenario into two cases. We consider $\tau = \kappa T$ [Figs. 3(b,e,h) with $\kappa = \frac{1}{2}$], where the lengths of $\mathbf{X}$ and Y remain proportional to one another. In this case, the T-duality seems to persist for all three dynamics. However, when $\tau = T - \xi$ [Figs. 3(c,f,i) with $\xi = 5$] where the size of Y remains fixed and $\mathbf{X}$ grows linearly with T, the T-duality is no longer observed, except for the Glauber dynamics. It is important to note that, for small ξ, the partial reconstructability coefficient $U(G \mid \mathbf{X}_{\text{future}}; \mathbf{X}_{\text{past}})$ becomes numerically unstable since both $I(\mathbf{X}_{\text{future}}; G \mid \mathbf{X}_{\text{past}})$ and $H(G \mid \mathbf{X}_{\text{past}})$ tend to zero. This is why the curves are much noisier in that case. Informed by these examples, we make the following conjecture:

Conjecture 1. *Let* $\mathbf{X} = (\mathbf{X}_{\text{past}}, \mathbf{X}_{\text{future}})$ *be a Markov chain, composed of the two consecutive Markov chains* $\mathbf{X}_{\text{past}}$ *and* $\mathbf{X}_{\text{future}}$ *of respective length* τ *and* $T - \tau$*, both conditioned on a discrete random variable* G*. Then, there exists a function* $g(T)$ *such that, if* τ *is dominated by* $g(T)$*, the partial uncertainty coefficients* $U(\mathbf{X}_{\text{future}} \mid G; \mathbf{X}_{\text{past}})$ *and* $U(G \mid \mathbf{X}_{\text{future}}; \mathbf{X}_{\text{past}})$ *are* T*-dual, and they are not otherwise.*

Supplementary Note VIII: Evaluation and bias of the mutual information in large systems

A. Effect of biased mutual information over the uncertainty coefficients

When an estimation of the mutual information is biased, it necessarily follows that an estimation of the resulting uncertainty coefficients will also be biased. Fortunately, we can show that the direction of the bias does not change either for the reconstructability $U(G \mid \mathbf{X})$ or the predictability $U(\mathbf{X} \mid G)$. Suppose that $\mathcal{I}_\varepsilon = I(\mathbf{X}; G)(1+\varepsilon)$ is an estimator of the mutual information, where $\varepsilon \in \mathbb{R}$ is a small bias which can be either positive or negative. Then, the corresponding estimators of the uncertainty coefficients, that we denote $\mathcal{P}_\varepsilon$ and $\mathcal{R}_\varepsilon$ for the predictability and the reconstructability,

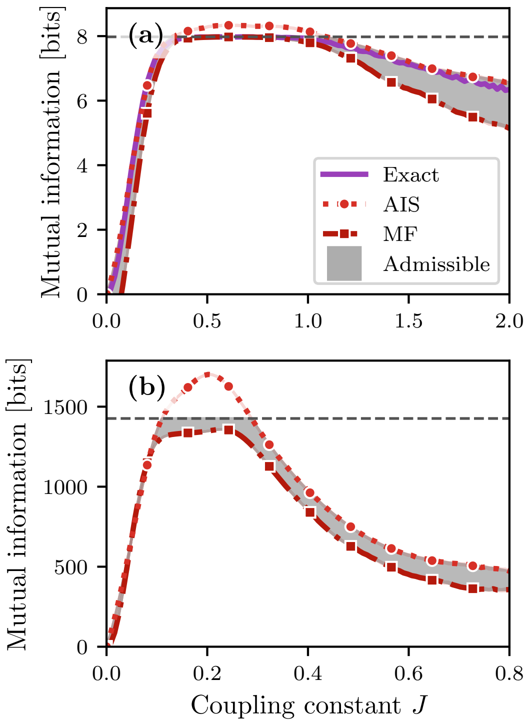

Supplementary Figure 4. **Estimators of the mutual information in the Glauber dynamics on Erdős-Rényi graphs as a function of the normalized coupling parameter** $J\langle k\rangle$: (a) $N = 5$, $E = 5$ and $T = 100$ (b) $N = 100$, $E = 250$ and $T = 1000$. The solid line in (a) corresponds to the exact evaluation of $I(\mathbf{X}; G)$ and is the same line as the one in Fig. 5(a). The circles and square in both (a) and (b) represent the values of $I(\mathbf{X}; G)$ computed using the AIS and the MF estimators, respectively. The dashed line indicates the upper bound of $I(\mathbf{X}; G)$, i.e., $\max\{H(G), H(\mathbf{X})\}$. We also show with a gray area the admissible values of $I(\mathbf{X}; G)$ bounded by the biased MF and AIS estimators.

respectively, are

$$\mathcal{P}_\varepsilon = \frac{\mathcal{I}_\varepsilon}{H(\mathbf{X} \mid G) + \mathcal{I}_\varepsilon} \,. \tag{40}$$

and

$$\mathcal{R}_\varepsilon = \frac{\mathcal{I}_\varepsilon}{H(G)} = U(G \,|\, \mathbf{X})(1+\varepsilon) \,, \tag{41}$$

Note that we also suppose that $H(G)$ and $H(\mathbf{X} \mid G)$ are not affected by the bias ε. For the first expression, we consider the first-order development of $\mathcal{P}_\varepsilon$ with respect to ε:

$$\mathcal{P}_\varepsilon = U(\mathbf{X} \,|\, G) \left[1 + \Big(1 - U(\mathbf{X} \,|\, G)\Big)\varepsilon - \mathcal{O}\left(\varepsilon^2\right)\right] \,. \tag{42}$$

Indeed, given that $U(\mathbf{X} \,|\, G) \geq 0$, the leading biased term $\Big(1 - U(\mathbf{X} \,|\, G)\Big)\varepsilon$ must have the same sign as ε. The second expression clearly shows that the bias of $\mathcal{R}_\varepsilon$ is exactly given by ε. Therefore, both $\mathcal{P}_\varepsilon$ and $\mathcal{R}_\varepsilon$ retain the direction of bias of $\mathcal{I}_\varepsilon$.

The variational mean-field (MF) estimator presented in Section IV.D of the main paper is biased and bounds the mutual information from below. For this reason, it is necessary to validate the MF estimator with another estimator, that is either exact or that bounds $I(\mathbf{X}; G)$ from above. In doing so, we can estimate the gap between the MF lower bound and the upper bound to assess the magnitude of the bias. In the next section, we present one such upper bound estimator.

B. Upper bound: The stepping-stone algorithm

Whereas the MF estimator represents a biased estimator of the posterior probability $P(G \mid \mathbf{X})$, there exists other Markov chain Monte-Carlo (MCMC) techniques that tackle the problem of estimating the evidence probability directly. The one we consider in this paper is obtained from an *annealed importance sampling* (AIS) procedure called the stepping-stone (SS) algorithm [12].

The procedure of the stepping-stone algorithm takes advantage of the fact that it is possible to sample efficiently from the posterior distribution $P(G \mid \mathbf{X})$ using MCMC (see main text). In order to compute an accurate estimator of the evidence probability $P(\mathbf{X})$, the procedure samples the space $\mathcal{G}$ according to $P_\beta(G \mid \mathbf{X})$, where $0 \leq \beta \leq 1$ is an inverse temperature parameter that dampens the influence of the likelihood such that

$$P_\beta(G \mid \mathbf{X}) \propto [P(\mathbf{X} \mid G)]^\beta P(G) \,. \tag{43}$$

The inverse temperature basically allows the Markov chain to navigate $\mathcal{G}$ efficiently to construct an accurate estimator of $P(\mathbf{X})$, that is where the graph samples are not all too close or too far

from the maximum posterior. More specifically, the AIS estimator is defined by

$$P_{\text{AIS}}(\mathbf{X}) = \prod_{k=1}^{K} \left\langle [P(\mathbf{X} \mid G_k)]^{\beta_k - \beta_{k-1}} \right\rangle , \tag{44}$$

where $0 = \beta_0 < \cdots < \beta_{K-1} = 1$ and the expectation is evaluated with respect to $G_k \sim P_{\beta_k}(G \mid \mathbf{X})$, for each k. Similarly to the mean-field estimator, we estimate this expectation by collect a sample $\mathcal{Q}_k^{(m)}$ of Q graphs distributed according to $P_{\beta_k}(G \mid \mathbf{X} = \mathbf{x}^{(m)})$, for each k.

Taking the log of this equation gives us an estimator of the log-evidence probability, which we can use to compute the mutual information directly:

$$\log P_{\text{AIS}}(\mathbf{X}) = \sum_{k=1}^{K} \log \left\{ \left\langle [P(\mathbf{X} \mid G = G_k)]^{\beta_k - \beta_{k-1}} \right\rangle \right\} . \tag{45}$$

Although the estimator for P_{AIS} is unbiased, the one for the log-evidence probability introduces a bias:

$$\log P(\mathbf{X}) \geq \log P_{\text{AIS}}(\mathbf{X}) . \tag{46}$$

This bias can be arbitrarily reduced by increasing K [12], although we found that doing so provides diminishing returns. Using the AIS estimator of the evidence probability, we obtain an AIS estimator of the mutual information such that

$$\begin{aligned} I(\mathbf{X}; G) \leq \frac{1}{M} \sum_{m=1}^{M} \Big[& \log P\Big(\mathbf{X} = \mathbf{x}^{(m)} \mid G = g^{(m)}\Big) \\ & - \log P_{\text{AIS}}\Big(\mathbf{X} = \mathbf{x}^{(m)}\Big)\Big] . \end{aligned} \tag{47}$$

Following Ref. [12], we use values of β_k distributed according to a beta distribution $\text{Beta}(\alpha, 1)$, where $\beta_k = \left(\frac{k}{K}\right)^{1/\alpha}$, such that increasing α controls how skewed around zero the sequence $\{\beta_k\}_{k=1..K}$ is. For Fig. 4, we fix $\alpha = 0.5$ and $K = 20$ and, for each value of β_k, we sample 1000 graphs from $P_{\beta_k}(G \mid \mathbf{X})$, proposing $5N$ moves in-between each sample (see main text).

C. Numerical results

Figure 4(a) shows the behavior of $I(\mathbf{X}; G)$ in the Glauber dynamics on a small Erdős-Rényi random graph as approximated using the MF and AIS estimators, and compares them to an exact evaluation based on an explicit graph enumeration used in Fig. 5. As expected the two estimators provide a lower and an upper bound for $I(\mathbf{X}; G)$, and these bounds are fairly tight.

Several caveats are in order. On the one hand, the bias of the AIS estimator can, in principle, be reduced arbitrarily by increasing the number K of temperature steps, but its evaluation becomes

quickly computationally costly. On the other hand, the evaluation of MF estimator is comparatively quicker, but cannot be improved by further sampling. The AIS estimator is accordingly closer to the exact value throughout, but it can sometimes overestimate the mutual information above its upper bound since $H(\mathbf{X})$ is overestimated while $H(\mathbf{X} \mid G)$ is not. The MF estimator can also yield negative values of $I(\mathbf{X}; G)$ for small values of J—i.e., regimes where $H(G \mid \mathbf{X}) \simeq H(G)$—due to an overestimated $H(G \mid \mathbf{X})$ becoming larger than $H(G)$.

Figure 4(b) shows the same experiment as in Fig. 4(a) but with larger graphs of $N = 100$ vertices and leads to similar observations: the AIS estimator is always greater than the MF estimator, and both estimators sometimes yields approximated values for $I(\mathbf{X}; G)$ outside of the valid range $[0, \max\{H(G), H(\mathbf{X})\}]$. Interestingly, these bounds are nevertheless fairly close to one another, as in the case $N = 5$.

Supplementary Note IX: Numerical estimation of the phase transition thresholds

We evaluate the phase transition thresholds of each dynamics using standard finite-size scaling techniques and Monte Carlo simulations (see Fig. 5). For Glauber, an adequate order parameter to visualize the phase transition is the magnetization $M := \frac{1}{NT}\sum_{i,t}|2X_{i,t}-1|$, where the absolute value breaks the spin symmetry [13]. In this process, it is well known that the susceptibility of the order parameter M, given by

$$\chi_M = \frac{\langle M^2\rangle - \langle M\rangle^2}{\langle M\rangle}, \tag{48}$$

diverges at the threshold $J = J_c$ of the phase transition for infinite size systems [13]. In finite systems, χ_M instead reaches a maximum at $J = J_c$. We use this fact to locate J_c and show the

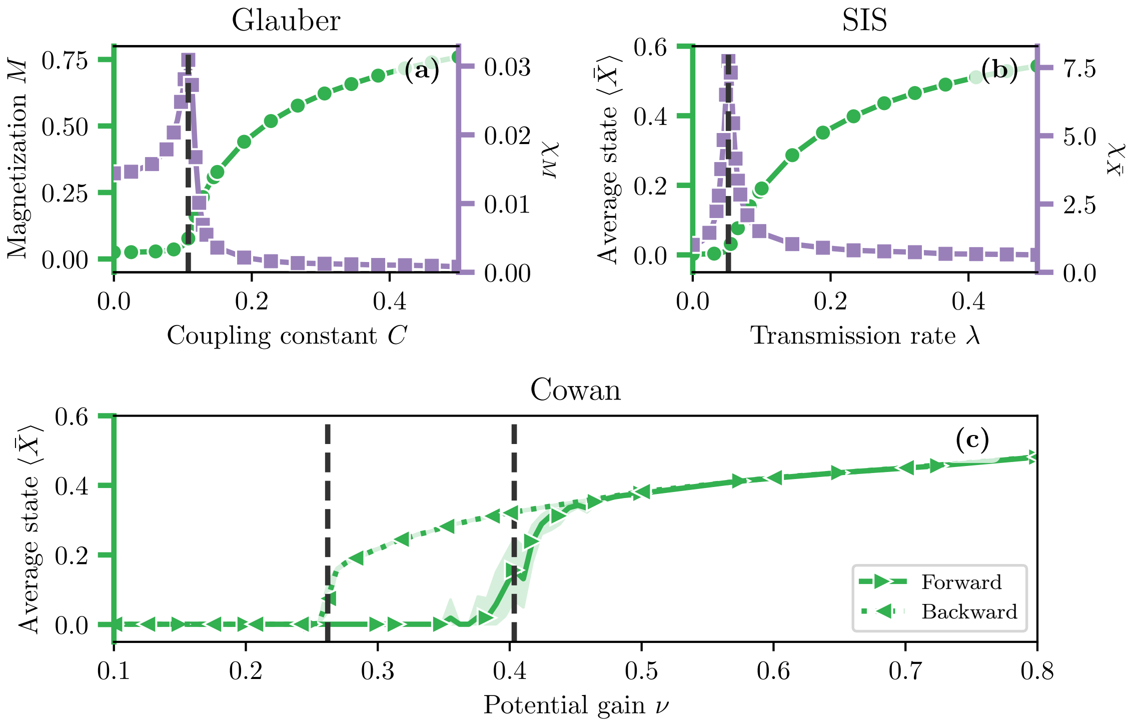

Supplementary Figure 5. Numerical evaluation of the phase transition thresholds: (a) Glauber dynamics, (b) SIS dynamics, (c) Cowan dynamics. For panels (a) and (b), the left axis (green) shows the order parameter (green circles), and the right axis (purple) shows the susceptibility (purple squares). For panel (c), only the order parameter is shown but for both the forward (right triangle) and backward (left triangle) branches. The values of the thresholds are indicated by the vertical dashed lines. We used the same parameters as those of Fig. 7 of the main paper, but increased the number of steps $T = 10^4$ to better sample from the dynamics. Each marker has been average over 48 realizations.

corresponding results in Fig. 5(a).

For the SIS dynamics, a similar finite-size scaling analysis can be carried out, but a suitable order parameter is rather the average state $\bar{\mathbf{X}} := \frac{1}{NT}\sum_{i,t} X_{i,t}$. We also use a definition of the susceptibility that is more convenient for spreading processes [14], given in terms of $\bar{\mathbf{X}}$:

$$\chi_{\bar{\mathbf{X}}} = \frac{\left\langle \bar{\mathbf{X}}^2 \right\rangle - \left\langle \bar{\mathbf{X}} \right\rangle^2}{\left\langle \bar{\mathbf{X}} \right\rangle}\,, \tag{49}$$

which also diverges at the phase transition threshold $\lambda = \lambda_c$ for infinite size systems. We show the results for SIS in Fig. 5(b).

Finally, for the Cowan dynamics, we have a first-order phase transition characterized by a discontinuity of the order parameter $\bar{\mathbf{X}}$ in the infinite size limit, and a bistable region bounded by two thresholds $\nu_c^b < \nu_c^f$. To find these two thresholds, we evaluate the order parameter $\bar{\mathbf{X}}$ for varying values of the parameter ν, and find the location where the discontinuity occurs. We obtain the forward and backward branches by using different initial conditions, where the system is nearly inactive—with one active vertex—and completely active—with no inactive vertex—, respectively.

For the Cowan dynamics, it is important to mention that since we consider relatively small systems ($N = 1000$ vertices), the bistable region is not clearly defined. Hence, a system starting in the forward branch can jump on the backward branch with a non-zero probability. This is why the expected discontinuity at the threshold is, in fact, populated (see Fig. 5(c)). This finite-size effect should be reduced for considering larger systems, but increasing N is unfortunately too computationally costly at the moment. Hence, to get a reasonable estimation of the thresholds in this scenario, we uniformly sample the set of ν's, compute $\left\langle \bar{\mathbf{X}} \right\rangle$ for all values of ν and find the point ν^* corresponding to the maximum gap between two points. Then, to increase the precision or this estimation, we zoom on a region centered at ν^* and do it again, until it converges. This method provides reasonably accurate thresholds for our purposes.

Supplementary References

[1] E. Laurence, N. Doyon, L. J. Dubé, and P. Desrosiers, "Spectral dimension reduction of complex dynamical networks," Phys. Rev. X **9**, 011042 (2019).

[2] H. Sanhedrai, J. Gao, A. Bashan, M. Schwartz, S. Havlin, and B. Barzel, "Reviving a failed network through microscopic interventions," Nat. Phys. **18**, 338–349 (2022).

[3] B. Prasse and P. Van Mieghem, "Predicting network dynamics without requiring the knowledge of the interaction graph," Proc. Natl. Acad. Sci. U.S.A. **119**, e2205517119 (2022).

[4] M. Vegué, V. Thibeault, P. Desrosiers, and A. Allard, "Dimension reduction of dynamics on modular and heterogeneous directed networks," PNAS nexus **2**, pgad150 (2023).

[5] T. M. Cover and J. A. Thomas, *Elements of Information Theory*, 2nd ed. (Wiley-Interscience, 2006).

[6] P. J. Davis, *Interpolation and approximation* (Dover, 1975).

[7] W. Rudin, *Real and complex analysis*, 3rd ed. (McGraw-Hill, 1986).

[8] G. E. Andrews, R. Askey, R. Roy, and R. Askey, *Special functions*, Vol. 71 (Cambridge University Press, 1999).

[9] F. N. Fritsch and J. Butland, "A method for constructing local monotone piecewise cubic interpolants," SIAM J. Sci. Stat. Comput. **5**, 300 (1984).

[10] G. Wolberg and I. Alfy, "An energy-minimization framework for monotonic cubic spline interpolation," J. Comput. Appl. Math. **143**, 145 (2002).

[11] J. Yao and K. E. Nelson, "An unconditionally monotone C^2 quartic spline method with nonoscillation derivatives," Advances in Pure Mathematics **8**, 25 (2018).

[12] W. Xie, P. O. Lewis, Y. Fan, L. Kuo, and M.-H. Chen, "Improving marginal likelihood estimation for bayesian phylogenetic model selection," Syst. Biol. **60**, 150 (2011).

[13] K. Binder and D. Heermann, *Monte Carlo Simulation in Statistical Physics* (Springer, 2010).

[14] S. C. Ferreira, C. Castellano, and R. Pastor-Satorras, "Epidemic thresholds of the susceptible-infected-susceptible model on networks: A comparison of numerical and theoretical results," Phys. Rev. E **86**, 041125 (2012).
